# The Austrian Capital, as of Interest to the American Medical Student

**Published:** 1890-01

**Authors:** Hugh Hagan

**Affiliations:** Vienna


### THE AUSTRIAN CAPITAL, AS OF INTEREST TO THE AMERICAN MEDICAL STUDENT.

VIENNA, NOV. 9, 89.

For years, it was my dream of all earthly happiness, to have just one year at the Vienna School of Medicine, and now that I am here, and realizing my dream, I will try and tell of some of the many advantages, from both a medical and surgical standpoint.

But first, let's have a look at the conditions and requirements.

The medical School is under the rule of the Royal University, and it is necessary that the applicant for matriculation first register with the dean of the medical department, then he registers his name, place of birth, nationality, religion, past scholastic relationships, and those branches which he will study. After having done this, he goes to the clerk's office, has his name and branches entered; then next to the cashier and pays the required fees, which are quite moderate. For instance, the fee for a didactic course, in general medicine, for the semester, is only 15 florins, or $6.00 of our money.

For practical courses, lasting from six to eight weeks, with 5 lectures a week, the prices range from 15 to fifty florins.

After having paid his required fees, he emerges from the University, and asks some one to show him to the Allgemeina Kranken Haus, or in other words, to the *General Austrian Hospital.*

This is situated on the Alserstrasse, and has a frontage of about 1000 ft. on this street, with about five wings the same length, and divided off into 9 courts each. The buildings are two and three stories high. The whole architecture is of the old German type, being stucco, and tile roofed.

This enclosure, or rather a net-work of buildings, contains over three thousand beds, the majority of which are always filled. The patients come from all parts of the empire, and each one must pay the sum of 40 kreuzer, or 20 cts. daily.

Here one will find clinical advantages, equaled nowhere. This is truly for the disciples of Hippocrates and Galen, an ideal hunting ground. For it matters not the disease, here it can be found in most perfect types.

The lectures are given in the Horsalle or lecture room, which is situated at the end of the ward set aside for that branch. The clinics are either below the lecture rooms, or in the wards, and here one not only gets the advantage of the experience of the professors, but can see for himself, the patient, and personally examine the same.

The wards are divided off for each special branch, thus, there are separate departments for the eye, ear, throat, nose, chest, internal medicine, surgery, nervous diseases, mental troubles, mostly acute, obstetrics, gynecology. This is a signal advantage over the other European schools and clinics, for in London, Paris and Berlin the student loses half his day in roaming from one hospital to another.

The lectures, given five times weekly, are mostly of two hours duration, the first hour being didactic, the second clinical.

Of such lectures one never tires, and to hear Prof. Billroth and Albert on surgery; Meynert on the diseases of the mind and nervous system; Carl Braun on obstetrics and gynecology; Kaposi on the skin diseases; Politzer on the ear; Fuchs on the eye, and thus to the end of the chapter, the student becomes at once an enthusiast.

The more practical courses, such as operative surgery, operations on the eye, ear, throat, &c. are given by the private docents, who are graduates, and have spent 6 years in the hospital.

These private courses are the ones mostly pursued by the post-graduate student; one can easily, in the course of two semesters, qualify oneself in one or more specialties. These courses are given for 15 florins, or $6.00, or for the more difficult the prices range a little higher.

The average cost of the two semesters, and special courses, ranges from $100.00 to $150.00. The pensions give board for $20.00 to $30.00, and books and instruments are to be had at about one half the price as in America. Thus we see, that with moderate economy, one could easily have the benefits of an ocean voyage, some European travel, and qualifications in one,s profession for about $1000.00, which sum any man would do well to invest, even from a most business like point of view.

Now just let's look in upon one or two lectures. Suppose we take a look at Prof. Billroth, on surgery, and Kaposi on the skin, and *see* what we can *see.*

At 8 o'clock, A. M., sharp, Prof. Kaposi enters. He is a tiny little fellow, with very bright eyes, and short beard, and quick though distinct speech, and few who have any knowledge of German, find difficulty in understanding him. Here one can see on Tuesday, more Lupus than, I will almost venture to say, exists in the Southern States. The Prof. uses the sharp spoon, and nitrate of silver to such a degree that the student opens his eyes in wonder.

Here one can during the week's lectures, see the most wonderful and varied types of skin diseases. Thus, for instance, last week he presented a case of small pox, two of leprosy, two of lichen rubroi, no end of eczemas, psoriasis, lupus, and more parasitic skin troubles, than one cares to see.

This keeps up, as not only do the hospital patients feed this clinic, but there are about 2000 walking patients who come daily to the different departments.

The professor is busy experimenting with some formula for the inflammatory skin affections; but, as yet, has not given the formula to the class.

Prof. Billroth, who looks much the worse from his recent illness, is punctual to the hour, and at 10, A. M., he enters with his genial smile, and after greeting the class, proceeds to discuss whatever subject he may have appointed. His classsical German is a treat, and one is completely oblivious of all else, save surgery, when he is talking.

The professor performs three or four operations daily, and as your turn comes, you are invited into the amphitheatre. Prof. Billroth is very perfect in his antisepsis, and uses the permanganate of potash, 1-1000, as his fluid, and has a 1-1000 sol. of sulphate copper, for the hands and sponges. He thinks fewer evils arise from these than from carbolic acid, and the bichloride.

He is a wonderfully steady and cautious operator, some of our younger surgeons might call him slow, but he is certainly sure

I won't tire you with longer descriptions of clinics, of which these are only examples.

Every courtesy is extended to the American, and every one does what he can to make the stranger's stay profitable and pleasant.

Now one word about the Polyclinic. Although not so well known as the Vienna Medical school, is very efficient, and has very extensive and well equipped laboratories for histology, pathology and hacteriology. Here, as in the regular college, one gets practical work, and at about the same price.

Hoping that more American medical students and doctors may take advantage of these wonderful opportunities, and that the SOUTHERN MEDICAL RECORD may have unprecedented success, as I know it will deserve,

I am yours truly,

HUGH HAGAN, M. D.

